# Causal Inference for Cross-Modal Action Selection: A Computational Study in a Decision Making Framework

**DOI:** 10.3389/fncom.2016.00062

**Published:** 2016-06-23

**Authors:** Mehdi Daemi, Laurence R. Harris, J. Douglas Crawford

**Affiliations:** ^1^Department of Biology and Neuroscience Graduate Diploma, York UniversityToronto, ON, Canada; ^2^Centre for Vision Research, York UniversityToronto, ON, Canada; ^3^Canadian Action and Perception NetworkToronto, ON, Canada; ^4^Department of Psychology, York UniversityToronto, ON, Canada; ^5^School of Kinesiology and Health Sciences, York UniversityToronto, ON, Canada; ^6^NSERC Brain and Action Program, York UniversityToronto, Canada

**Keywords:** causal inference, decision-making, multisensory integration, working memory, spatiotemporal similarity, saliency map of space, report of sameness

## Abstract

Animals try to make sense of sensory information from multiple modalities by categorizing them into perceptions of individual or multiple external objects or internal concepts. For example, the brain constructs sensory, spatial representations of the locations of visual and auditory stimuli in the visual and auditory cortices based on retinal and cochlear stimulations. Currently, it is not known how the brain compares the temporal and spatial features of these sensory representations to decide whether they originate from the same or separate sources in space. Here, we propose a computational model of how the brain might solve such a task. We reduce the visual and auditory information to time-varying, finite-dimensional signals. We introduce controlled, leaky integrators as working memory that retains the sensory information for the limited time-course of task implementation. We propose our model within an evidence-based, decision-making framework, where the alternative plan units are saliency maps of space. A spatiotemporal similarity measure, computed directly from the unimodal signals, is suggested as the criterion to infer common or separate causes. We provide simulations that (1) validate our model against behavioral, experimental results in tasks where the participants were asked to report common or separate causes for cross-modal stimuli presented with arbitrary spatial and temporal disparities. (2) Predict the behavior in novel experiments where stimuli have different combinations of spatial, temporal, and reliability features. (3) Illustrate the dynamics of the proposed internal system. These results confirm our spatiotemporal similarity measure as a viable criterion for causal inference, and our decision-making framework as a viable mechanism for target selection, which may be used by the brain in cross-modal situations. Further, we suggest that a similar approach can be extended to other cognitive problems where working memory is a limiting factor, such as target selection among higher numbers of stimuli and selections among other modality combinations.

## Introduction

Sensory systems detect different types of signals originating from objects in the surrounding environment. For example, visual information is carried by electromagnetic waves with a specific range of frequencies, whereas auditory information is carried by mechanical waves with a certain range of frequencies. Our brain constructs various perceptions and plans various actions in space and time, which can be triggered by sensations from multiple modalities. Integration of multimodal sensory information has been studied for temporal perceptions, e.g., perception of duration (Burr et al., 2009; Klink et al., 2011) and simultaneity (Harrar and Harris, 2008; Virsu et al., 2008), for spatial perceptions, e.g., spatial localization (Alais and Burr, 2004) and motion direction perception (Sadaghiani et al., 2009), for causal inference (Slutsky and Recanzone, 2001; Wallace et al., 2004), and also for action (Frens et al., 1995; Van Wanrooij et al., 2009). Here we are concerned with how the multisensory information is processed for causal inference.

Causal inference in animals is the process of estimating what events in outside world has caused the sensory representations in the brain (Shams and Beierholm, 2010; Lochmann and Deneve, 2011). In presence of multiple sensory representations, we compare their features to infer if they have a unique cause or not. A commonly studied case is when visual and auditory information is used to construct spatial and temporal perceptual features. If the temporal features are similar to each other, a common cause may be perceived overriding mismatches in their spatial features (Vroomen et al., 2001a,b; Godfroy et al., 2003). Similarly, if the spatial features are similar to each other, a common cause may again be perceived despite mismatches between temporal features (Vroomen and Keetels, 2006; Vroomen and Stekelenburg, 2011). These spatial and temporal binding effects break down at large spatial or temporal disparities (Slutsky and Recanzone, 2001; Wallace et al., 2004). In this paper we intend to propose a unique mechanism for causal inference which explains all this seemingly disparate evidence. However, let's first review some previous attempts on solving this problem.

In one study (Alais and Burr, 2004), observers were asked to report the location of a stimulus consisting of a flash and click presented with a spatial conflict. The spatial reliability of the visual signal was varied. The participants were told that the flash and click belonged to a unique object. For the case of the most conspicuous visual stimuli they observed the classical ventriloquist effect such that the participants perceive the object close to the position of the visual stimulus. For heavily blurred visual stimuli, they perceive the object close to the auditory stimulus. For intermediate levels of blurriness, they perceive the object somewhere between the positions of the visual and auditory stimuli. Their results imply that, when the observers assume a common cause for the cross-modal stimuli, an intermediate position closer to the more reliable of the stimuli, is perceived as the location of the common cause. This idea was modeled, assuming Gaussian distributions for the unisensory cues, by Bayesian integration of the distributions, leading to the average of the two position cues weighted by the inverse of the variances of their distributions (Alais and Burr, 2004). Others tried to implement this optimal integration by a single-neuron model (Patton and Anastasio, 2003) or a model of a population of neurons (Ma et al., 2006).

Other experimental studies let the participants decide whether two cross-modal signals belonged to a unique object or not (Slutsky and Recanzone, 2001; Wallace et al., 2004). Such studies changed the spatial and temporal relationships between the two stimuli. For very short-duration and synchronous stimuli, the participants reported a unique object as the source of the signals and perceived it at the weighted average of the position of the two signals. When the presentation time was extended or temporal disparity was introduced between the signals, the chance of reporting a unique cause for two spatially disparate signals decreased drastically. Also for synchronous stimuli, increasing the spatial disparity between the stimuli decreased the percentage of the trials in which the participants reported a unique object as the source. Their results showed that when participants are not told to assume a common source for the stimuli, they might localize the stimuli in common or separate spatial positions depending on the spatial and temporal features of the stimuli.

Some theoretical studies have tried to model the effect of spatial disparity (Hairston et al., 2003) on the report of a common cause (Körding et al., 2007; Sato et al., 2007). However, these studies ignored the temporal effect. They used the uncertain spatial cues, detected through multiple sensory channels, to calculate the probabilities of them arising from same or separate sources. If the same source is more likely, these models calculate the optimal estimate of the location of the same source as the weighted average of the cues. If separate sources are more likely, the models shown that the uncertain spatial cues are the best estimates of the two locations. A physiologically realistic framework for these models has not been offered (Ma and Rahmati, 2013). Some other theoretical studies reduce the criterion for fusion to the temporal features of the events, ignore the spatial disparity, and propose that the cross-modal events are bound together if they happen within a relative time window (Colonius and Diederich, 2010; Diederich and Colonius, 2015).

Here we want to propose a more general approach which considers the spatial and temporal dimensions in a common framework. We suggest a model of how the brain solves the causal inference problem for spatial localization for cross-modal, audiovisual stimuli with arbitrary spatial and temporal disparities. We propose the model at the computational level (Marr, 1982), not assuming a specific probability distribution or neural representation for the spatial position of the stimuli. We consider two stimuli, visual and auditory, with only spatial and temporal features. However, other problems with more than two stimuli, or with other modality combinations, or with stimuli of semantic or emotional significance can also be tackled by our approach. We consider the stimuli to be composed of multidimensional, time-varying, position signals which communicate the time and place of the stimuli. Our model is proposed within an evidence-based decision making framework including a short-term memory, in the form of a leaky integrator, and a spatiotemporal similarity measure as the criterion for inferring the cause of the input signals. The short-term memory retains spatial information (not information about the order and temporal interrelations of events) and our similarity measure captures spatial and temporal disparities between the stimuli (not a higher-level order relation between them in time or space). We use this model to simulate known psychophysical results, and to generate predictions that can be used to test the model. Such results demonstrate that a model constructed in a decision making framework and inferring a causal structure based on a spatiotemporal similarity measure explains the behavioral results and could possibly be used by the brain to solve the target selection problem when cross-modal stimuli are presented.

## Model overview

The problem we are addressing is causal inference in localization of cross-modal stimuli in which the spatiotemporal properties of the components vary. To solve this problem, we borrow two popular concepts from cognitive neuroscience that (perhaps surprisingly) have not yet been incorporated into models of multisensory spatial integration: decision making (Wang, 2008), and working memory (Baddeley, 2003b). Although the computations in this model could pertain to any cognitive or behavioral use of causal inference from multimodal inputs, we designed this model with output to the gaze control system in mind, because this is one of the best understood systems in the brain (Bell et al., 2005) and because numerous gaze-control laboratories are capable of testing our predictions. Thus, one can think of the output of the model as dictating whether a gaze-shift will be made toward the visual stimulus, the auditory stimulus, or a combined representation derived from both. Finally, we have arranged the general order and nature of our model algorithms to be compatible with the known biology of these systems but focus the current study on replicating and predicting psychophysical results.

**Figure 1:**
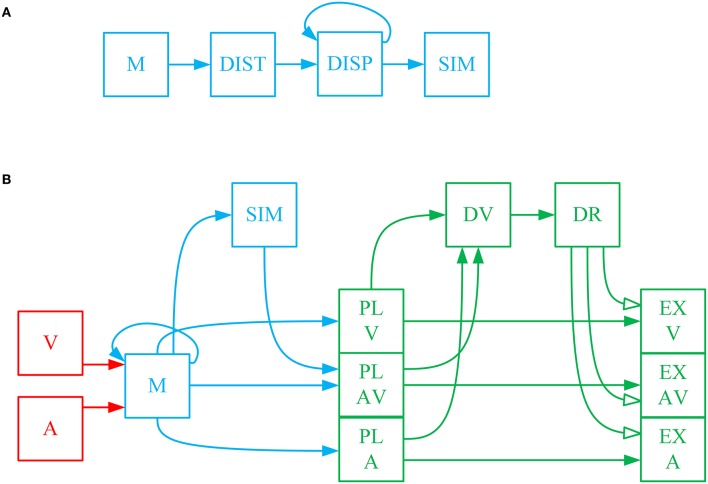
**(A)** Computation of the spatiotemporal similarity measure and using it to make a call on the sameness of the cause of cross-modal signals. The eye-centered, spatial components of the visual and auditory signals, which are stored in short-term memory ($\vec{M}$) are fed into the unit $\vec{DIST}$ to calculate the spatial distance between them as a function of time. The spatial distance is then sent to the unit $\vec{DISP}$, called spatiotemporal disparity, where it is integrated across time. The spatiotemporal disparity is then sent to the unit *SIM*, called spatiotemporal similarity measure, where is goes through an inverting and normalizing function. The spatiotemporal similarity measure is used for making a call on the sameness of the cause of the two received signals. This is done by sending the similarity measure to the unit *SAM*, called sameness call, where a threshold function is applied to it. **(B)** The complete model of gaze-shift, target selection in cross-modal situations. The visual ($\vec{V}$) and auditory ($\vec{A}$) signals are stored in a multisensory memory ($\vec{M}$) structure. In parallel, the visual and auditory signals are used for computation of spatiotemporal similarity measure (*SIM*), as illustrated in detail in **(A)**. The three alternative plans are constructed as saliency maps from the memorized information and are represented initially in the plan layer in three units $\vec{PL\_V}$, $\vec{PL\_A}$, and $\vec{PL\_AV}$. The unisensory plans are the unisensory stimulus positions along with their reliabilities which are regarded as equivalent to their saliencies. The multisensory plan is the weighted average (by reliabilities) position of the cross-modal stimuli along with the similarity measure as its saliency. The decision variable is constructed in the unit $\vec{DV}$ by communicating the saliencies of the three plans. The result of the decision is computed in $\vec{DR}$ by a function which implements the idea that the multisensory plan wins if the similarity measure is greater than a threshold and the more reliable of the unisensory plans wins if the similarity measure is lower than the threshold. The spatial components of the three plans are communicated from the plan layer to three units $\vec{EX\_V}$, $\vec{EX\_A}$, and $\vec{EX\_AV}$ in the execution layer. The result of the decision is materialized by selective inhibition of the plan representations in the execution layer. Only the winning plan is disinhibited, based on the decision result, and is sent for execution.

The sensory information received from stimuli in the environment is transient as most stimuli are only present for a limited time. Sensory information about the position and reliability of multimodal stimuli is moved to, and temporarily stored in, working memory where operations such as integration and computation of similarity take place. Working memory, in general, is used to bring together different pieces of information for cognitive processing with the goal of performing tasks such as reasoning, problem solving or action planning (D'Esposito et al., 1995; Baddeley, 2003a). Working memory is a distributed system in the brain, with multiple brain areas activated depending on the specific task being implemented (Courtney et al., 1997; Haxby et al., 2000; Fuster, 2004). Working memory in our model comprises four computational units (shown in blue in Figure 1) that are responsible for retaining sensory information, integrating spatial cues, computing a similarity measure, and feeding the decision-making circuitry (Bechara et al., 1998).

We propose our model within a decision-making framework. Decision making is the process of deliberation resulting in the commitment to one of multiple alternative plans (Gold and Shadlen, 2007; Heekeren et al., 2008; Cisek and Kalaska, 2010). The deliberative process consists in the accumulation of evidence through processing the available information. This is realized in the evolution of systemic decision variables through time. The result of the decision is determined by a rule which is applied to the decision variables. Decision rules determine how or when the decision variable is interpreted to arrive at a commitment to a particular plan (Churchland et al., 2008). The decision result is the output variable of the evidence accumulation and rule application, that determines which plan is to be executed. Accumulation of evidence changes the decision variables and may change the decision result (Bogacz, 2007). As we shall see, each of these features has been incorporated into our model (green in Figure 1B).

The first part of the decision is to decide whether there is a unique cause for the two signals or if they correspond to two separate events. As explained before, the experimental evidence shows that this decision is determined based on the spatial and temporal relationship between the cross-modal stimuli (Wallace et al., 2004). We propose a measure of spatiotemporal similarity between the two received signals that is used for making this decision. Figure 1A shows how this measure is calculated in working memory. The spatiotemporal pattern of stimuli presentation is captured in a temporally changing spatial position signal, decoded from the representations of sensory space in the brain. Spatial distance (*DIST*) between the two stimuli, as a function of time, is first calculated. Spatial distance is integrated through time to calculate the spatiotemporal disparity ($\vec{DISP}$). Spatiotemporal similarity measure (*SIM*) is calculated by applying a function that inverts and normalizes the spatiotemporal disparity. This time-varying, similarity measure decreases with increases in the spatial disparity and/or temporal disparity between the two presented stimuli.

The complete problem can be conceptualized as choosing between three possible scenarios: (1) the signals are coming from one same object. In this case the target for gaze-shift is constructed as a weighted average of the visual and auditory estimates. (2) The signals are coming from different objects and the visual stimulus is more salient, in which case the target is chosen to be at the location of the visual stimulus. (3) The signals are coming from different objects and the auditory stimulus is more salient, so, the target is chosen to be at the location of the auditory stimulus. Thus, the main task for our model is to infer one of these three scenarios from a given pair of multisensory inputs.

The complete model is shown in Figure 1B. The inputs to the system are the temporally changing position signals of the visual and auditory stimuli along with their reliabilities ($\vec{V}$ and $\vec{A}$). These spatial position signals are temporarily stored in a memory structure ($\vec{M}$). The spatiotemporal similarity measure (*SIM*) is computed from the position signals stored in memory. The previously mentioned three possible scenarios are physically realized in the form of three plan representations. Each plan unit represents the potential goal for an attention shift (if that plan wins) along with the saliency of the plan. The visual ($\vec{PL\_V}$) and auditory ($\vec{PL\_A}$) plan units represent the position of the corresponding stimuli along with their reliabilities (Körding et al., 2007; Rowland et al., 2007; as our stimuli don't bear any emotional significance or semantic meaning, their saliency is reduced to their reliability). Reliability in our model is a one-dimensional, real-valued parameter, which can change between 0 and 1 for the least to most reliable, and is an input to the model. We presume that this reliability can be calculated, upstream of our model, based on the representation of the spatial position, e.g., the inverse of the variance for a normal distribution (Körding et al., 2007; Ohshiro et al., 2011). The multisensory plan ($\vec{PL\_AV}$) unit represents average of the positions of the two stimuli weighted in proportion to their respective reliabilities (Alais and Burr, 2004). The saliency of the multisensory plan is proposed to be the spatiotemporal similarity measure.

The decision variable ($\vec{DV}$) is constructed from the saliencies of the three alternative plans. The decision on same or separate causes for the signals is made by comparing the saliency of the multisensory plan with a threshold. We assume this threshold is tunable, and one possible way to account for the effects of emotional or semantic value of stimuli on sensory fusion is to be able to adjust this threshold. However, as this is beyond the scope of our model, we set the threshold to 0.5 (to match the experimental evidence, see below) and for consistency we use the same value for all of our predictive simulations. As long as saliency, i.e., the spatiotemporal similarity measure, is above threshold the decision that they are from the same source is preferred. If the similarity measure drops below threshold the decision changes to that they originate from separate sources. In this case, the decision concerning which cause forms the goal of a shift of attention is made by comparing the saliencies of the two unisensory plans. The overall result of this three-way decision ($\vec{DR}$) is stored as a 3-D signal that allows communication of only the winning plan to the execution units ($\vec{EX\_V}$, $\vec{EX\_A}$, $\vec{EX\_AV}$). This is implemented through the decision result. $\vec{DR}$ keeps all *EX* units under constant inhibition. When a plan wins, its corresponding *EX* unit is disinhibited.

The general outline of the model is inspired by known properties of the visual, auditory, and gaze control systems. The visual signal is the position of the visual stimulus in eye-centered coordinates (Andersen et al., 1997; Maier and Groh, 2009). Auditory space is encoded initially in a craniocentric frame of reference (Knudsen and Konishi, 1978; Knudsen and Knudsen, 1983) as the auditory receptors are fixed to the head. For multisensory information processing and motor planning, the two sensory signals, $\vec{V}$ and $\vec{A}$, should be in a common reference frame (Jay and Sparks, 1987; Andersen et al., 1997) which has been shown to be eye-centered for action involving the gaze-control system and early aspects of reach planning (Groh and Sparks, 1992; Cohen and Andersen, 2000; Pouget et al., 2002). The sensory signals are then sent to the distributed network of working memory. Posterior parietal and dorsolateral prefrontal cortex have been shown to actively maintain such signals (Funahashi et al., 1989; Cohen et al., 1997), similar to the short-term memory $\vec{M}$ in our model. The prefrontal cortex is involved in the higher-order, executive functions of working memory (D'Esposito and Postle, 2015), including integration of the signals into unique events, realized in our model through $\vec{DIST}$, $\vec{DISP}$, and $\vec{SIM}$. It is thought that the working memory then feeds the plan representations of the decision making circuitry in frontal cortex (Jones et al., 1977; Canteras et al., 1990; Berendse et al., 1992; Yeterian and Pandya, 1994; Levesque et al., 1996), like our plan representations in play layer *PL*. Plan representations are then thought to send bids, e.g., their saliencies as in our case, to a central arbitrating system (Redgrave et al., 1999), e.g., the telencephalic decision centers, that gate their access to effectors. This is represented in our model through $\vec{DV}$ and $\vec{DR}$ and their connection which realizes a decision rule. The basal ganglia are thought to receive the result of the decision from cortex (Beiser and Houk, 1998; Koós and Tepper, 1999; Gernert et al., 2000) and implement it through selective disinhibition of cortical channels, which is abstracted in our model through the multiplicative effect of the $\vec{DR}$ on plan representations in execution layer *EX*. In order to plan a gaze-shift, for example, the final winning plan is sent to the superior colliculus (Munoz and Guitton, 1989; Klier et al., 2001). This command could then be used to drive the eye-head coordination system (Klier et al., 2003; Daemi and Crawford, 2015) to reorient the line of sight to the appropriate target.

## Mathematical formulation

### Methods

Our model implements causal inference through a decision making network for planning actions in a dynamic environment. This contrasts to previous approaches which either described (1) inference as chains of if-then rules which statically transform the internal states of the system (Newell and Simon, 1972; Anderson, 1983) or (2) goal-directed motor planning within the time constraints of environmental interactions (van Gelder, 1998). While the former approach ignores the short-term dynamics of perception and action, the latter ignores the internal system, and sacrifices the high-level linguistic processes, such as complex planning and deductive reasoning. Our goal was to integrate both “dynamic perception/action” and “high-level inference” in a way consistent with our knowledge of human and animal cognitive systems (see Section Model Overview).

To do this, we adopt a unified approach where a model is identified by functions of both the internal state variables and the time. Inspired by the brain, such more general models are realized through a distributed network of parallel processing units. This approach simultaneously accounts for syntactic manipulations of representations underlying inference, and flexible control of information routing between different units through time (Eliasmith, 2013). Although we do not deal here with the neural implementation of the model, all the representations and transformations are designed based on the known neurophysiology, and can be neurally realized by a recent theoretical approach, neural engineering framework, which unifies the symbolic, connectionist, and dynamicist viewpoints (Eliasmith and Anderson, 2003; Eliasmith et al., 2012). The relatively high number of variables in such models is because we are modeling an adaptive, robust biological system which can behave and survive in an uncertain, changing environment.

More specifically, we implement an evidence-based decision making process, whose representations are evolving through time. The inference's syntactic manipulations are realized through selective inhibition of plan representations, as inspired by the brain. Routing the information through the system is realized in a unified architecture where all attractor networks are controlled integrators which include a dimension (controlled leak) whose value controls whether the structure updates its value by it input, retains its current value, or clears its content. Information routing is controlled by the dynamics of the system not by the choice of modeler, as it is in the brain. As a result, inference is realized through time, evolving as empirical evidence is accumulated, helping us to survive in a highly dynamic environment.

### Unisensory signals

When visual or auditory stimuli appear in the environment, they get detected at specific spatial locations, within specific time windows. The visual stimulus is encoded in retinal coordinates, i.e., an eye-centered frame of reference. The auditory stimulus is initially encoded relative to head, however, for cognitive and motor purposes, this code is transformed into an eye-centered reference frame as well (Maier and Groh, 2009). The unisensory input signals in our model are transient, time-varying, four-dimensional vectors. The four dimensions include a first component for existence of the signal, a second component for reliability of the signal and two last components for the eye-centered position of the signal in the spherical coordinates. The existence component gets value 1 or 0 based on whether or not a stimulus is detected in the environment, by stimulation of the sensory receptors. It controls the interaction of the sensory information with memory (explained next). The reliability component, changing between 0 and 1 for least to most reliable, is computed from the early representation of the signal (Körding et al., 2007; Ohshiro et al., 2011), and indicates how reliable the representation is about the position of the stimulus.

(1) $$\begin{array}{lll} \vec{V}\left(t\right) & = & \left[\begin{array}{c} ext_{v}\\ rel_{v}\\ ech_{v}\\ ecv_{v} \end{array}\right] \end{array}$$

(2) $$\begin{array}{lll} \vec{A}\left(t\right) & = & \left[\begin{array}{c} ext_{a}\\ rel_{a}\\ ech_{a}\\ ecv_{a} \end{array}\right] \end{array}$$

### Short-term memory

The transiently presented sensory signals need to be temporarily stored for further cognitive processing, e.g., inference (D'Esposito et al., 1995; Baddeley, 2003a), and then feeding the decision making circuitry. Accordingly, the unisensory signals are first communicated a short-term memory structure. It is a state space of finite dimensions which temporarily stores the unisensory signals in a unique representation. It consists of leaky integrators with controllable leaks. Sensory information is retained across eight dimensions of this state space, four dimensions for each modality. Those four modality-specific dimensions include a first component controlling the integrator's leak, and three components storing the last three dimensions of the unisensory signals:

(3) $$\begin{array}{l} \vec{M}\left(t\right)=\left[\begin{array}{c} lk_{mv}\\ rel_{mv}\\ ech_{mv}\\ ecv_{mv}\\ \begin{array}{c} lk_{ma}\\ rel_{ma}\\ ech_{ma}\\ ecv_{ma} \end{array} \end{array}\right] \end{array}$$

This memory structure, in connection with the transient sensory signals, is governed by these nonlinear state-space equations. In a general sense, such state space equations are the basis of constructing attractor neural networks which is believed to underlie memory structures in the brain (Conklin and Eliasmith, 2005; Singh and Eliasmith, 2006). The boundary and input conditions of these differential equations are dictated by a dynamic environment. Therefore, the current state of the state space is controlled internally, by the controllable leaks, in constant interaction with the environment. However, more specifically, before any input comes in, all dimensions of the state space are zero.

(4) $$\begin{array}{l} \left[\begin{array}{c} {l\overset{\cdot }{k}}_{mv}\\ {rėl}_{mv}\\ {ecḣ}_{mv}\\ {ec\overset{\cdot }{v}}_{mv}\\ {l\overset{\cdot }{k}}_{ma}\\ {re\overset{\cdot }{l}}_{ma}\\ {ecḣ}_{ma}\\ {ec\overset{\cdot }{v}}_{ma} \end{array}\right]=\left[\begin{array}{c} 00000000\\ 0\left(1-{lk}_{mv}\right)000000\\ 00\left(1-{lk}_{mv}\right)00000\\ 000\left(1-{lk}_{mv}\right)0000\\ 00000000\\ 00000\left(1-{lk}_{ma}\right)00\\ 000000\left(1-{lk}_{ma}\right)0\\ 0000000\left(1-{lk}_{ma}\right) \end{array}\right]\times \left[\begin{array}{c} {lk}_{mv}\\ {rel}_{mv}\\ {ech}_{mv}\\ {ecv}_{mv}\\ {lk}_{ma}\\ {rel}_{ma}\\ {ech}_{ma}\\ {ecv}_{ma} \end{array}\right]\\ \;+\left[\begin{array}{c} 10000000\\ 01000000\\ 00100000\\ 00010000\\ 00001000\\ 00000100\\ 00000010\\ 00000001 \end{array}\right]\times \left[\begin{array}{c} {ext}_{v}\\ {rel}_{v}\\ {ech}_{v}\\ {ecv}_{v}\\ {ext}_{a}\\ {rel}_{a}\\ {ech}_{a}\\ {ecv}_{a} \end{array}\right] \end{array}$$

The controllable leaks characterize the behavior of the controlled integrator (Table 1; Eliasmith, 2005). The two leaks are fed by the existence component of the corresponding sensory input. The existence component is 1 when the stimulus is present and is 0 when it is not, so the leaks always assume digital values 0 or 1. This means the integrator is updated by the new input when the input is present and maintains the current value when no input is present.

**Table 1:** **The effect of the leak on the behavior of a leaky integrator**.

	**Leak = 0**	**Leak = 1**
No input coming	Keeps the current value	Clears the memory
Input coming	Integrates and accumulates the input	Updates to the input

*Theoretically speaking, if the leak gets a value between 0 and 1, when there is no input the integrator clears the memory with a speed controlled by the leak, and when there is an input the integrator integrates the input with a speed controlled by the leak. However, both our integrator structures always assume digital values of 0 or 1*.

### Spatiotemporal similarity measure

The cognitive processing in working memory, in our model, consists of computing a measure of similarity between the two unisensory signals based on their spatial positions and temporal profiles. Figure 1A illustrates the connectivity of structures for calculating this measure. We start with the spatial distance *DIST*. The spatial distance between the two unisensory stimuli is calculated from the information stored in the short-term memory about the spatial positions of the stimuli. It is computed, in spherical coordinates, in the connection from $\vec{M}$ to *DIST*:

(5) $$\begin{array}{l} DIST\left(t\right)=\left[dist\right]=\cos^{-1}[\cos\left(\;ech_{mv}\right)\times \cos\left(\;ech_{ma}\right)\;\\ \;+\sin\left(ech_{mv}\right)\times \sin\left(ech_{ma}\right)\\ \;\times \cos\left(ecv_{mv}-\;ecv_{ma}\right)] \end{array}$$

The spatiotemporal disparity $\vec{DISP}$ is then calculated from the spatial distance by integrating it across time. Our proposed structure is a state space of two dimensions. This is, again, a leaky integrator with controllable leak. The two dimensions of this state space include a first component controlling the integrator's leak and a second component where the integrated value of the spatial distance is accumulated.

(6) $$\begin{array}{l} \vec{DISP}\left(t\right)=\;\left[\begin{array}{c} \;lk_{disp}\\ disp \end{array}\right] \end{array}$$

These state space equations characterize the behavior of this integrator. Before introduction of inputs, all dimensions of the state space are zero.

(7) $$\begin{array}{l} \left[\begin{array}{c} lk_{disp}\\ disp \end{array}\right]=\left[\begin{array}{cc} 0 & 0\\ 0 & \left(1-lk_{disp}\right) \end{array}\right]\times \left[\begin{array}{c} lk_{disp}\\ disp \end{array}\right]+\left[\begin{array}{c} 0\\ 1 \end{array}\right]\times \left[dist\right] \end{array}$$

Here, the leak does not need to be controlled based on existence of the input. The leak is internal to the functioning of the integrator, and represents a value 0 all through the stimulus presentation window. That is because we want it to integrate the input when there is any, and retain the current value when there is no input (Table 1). The result of this integration gives us a measure of spatiotemporal disparity between the visual and auditory stimuli. A tangent hyperbolic function is then applied on the disparity measure to calculate a measure of similarity between the two stimuli:

(8) $$\begin{array}{l} SIM\left(t\right)=\left[sim\right]=\;1\;-\text{ tanh }\left(0.5\times disp\right) \end{array}$$

This makes the similarity measure change between 0 and 1 for the least to the most similar. Equations in this section might not be supported by a known brain mechanism, however, we will later show that using spatiotemporal similarity as the criterion to infer unique or separate causes can explain the experimental evidence about the relation of such judgements with the spatial and temporal disparities between cross-modal stimuli.

### Decision making process

The information processed in working memory is then communicated to the decision making circuitry (Bechara et al., 1998), which realizes the causal inference in our model. We introduce three plan units, visual, auditory and multisensory, which are fed by the working memory. Each of these channels is a 3-dimensional vector whose first component represents the saliency of that plan. The saliency of each of the unisensory plans is reduced to its reliability. The last two components of the two unisensory plans represent their respective spatial positions as stored in short-term memory:

(9) $$\begin{array}{l} \vec{PL\text{\_}V}\left(t\right)=\left[\begin{array}{c} \;sal_{plv}\\ \;ech_{plv}\\ \;ecv_{plv} \end{array}\right]=\left[\begin{array}{c} rel_{mv}\\ \;ech_{mv}\\ \;ecv_{mv} \end{array}\right] \end{array}$$

(10) $$\begin{array}{l} \vec{PL\text{\_}A}\left(t\right)=\left[\begin{array}{c} \;sal_{pla}\\ \;ech_{pla}\\ \;ecv_{pla} \end{array}\right]=\left[\begin{array}{c} rel_{ma}\\ \;ech_{ma}\\ \;ecv_{ma} \end{array}\right] \end{array}$$

Integration of the unimodal signals, which might be used to drive a gaze-shift, is implemented in working memory, in its connection to multisensory plan representation. The multisensory channel represents a weighted average of the positions of the two stimuli, weighted by their reliabilities. The saliency of the multisensory plan is considered to be the spatiotemporal similarity between the two stimuli, which varies between 0, for least similar, and 1, for most similar:

(11) $$\begin{array}{l} \vec{PL\text{\_}AV}\left(t\right)=\left[\begin{array}{c} \;sal_{plav}\\ \;ech_{plav}\\ \;ecv_{plav} \end{array}\right]\\ \;=\left[\begin{array}{c} sim\\ \;rel_{mv}\times \;ech_{mv}+\;rel_{ma}\times \;ech_{ma}\\ \;rel_{mv}\times \;ecv_{mv}+\;rel_{ma}\times \;ecv_{ma} \end{array}\right] \end{array}$$

Now, we are ready to construct our decision variable, realizing a central decision center (Gold and Shadlen, 2007). We propose a three-dimensional vector as the decision variable $\vec{DV}$ which is completely characterized by the saliency of the plan (PL) representations:

(12) $$\begin{array}{l} \vec{DV}\left(t\right)=\;\left[\begin{array}{c} dv_{v}\\ dv_{a}\\ dv_{av} \end{array}\right]=\;\left[\begin{array}{c} sal_{plv}\\ sal_{pla}\\ sal_{plav} \end{array}\right] \end{array}$$

The values of the components of $\vec{DV}$ determine the decision about which of the visual, auditory or multisensory channels drives the final goal of gaze-shift. The result of this decision is to disinhibit the desired channel and keep inhibiting the undesired ones (explained below). The result of the decision making process is temporarily stored in another structure that we call ‘decision result’ or $\vec{DR}$. The decision function, which transforms $\vec{DV}$ to $\vec{DR}$, is the abstract underlying mechanism of inference in our model, and is formed through this idea:

(13) $$Decision\;Result=\;\{\begin{array}{cc} \left[\begin{array}{c} 1\\ 0\\ 0 \end{array}\right] & if\;sim>threshold\\ \left[\begin{array}{c} 0\\ 1\\ 0 \end{array}\right] & if\;sim<threshold\;and\;rel_{v}>\;rel_{a}\\ \left[\begin{array}{c} 0\\ 0\\ 1 \end{array}\right] & if\;sim<threshold\;and\;rel_{a}>\;rel_{v} \end{array}$$

Which is mathematically realized by this proposed functionality:

(14) $$\begin{array}{l} \vec{DR}\left(t\right)=\;\left[\begin{array}{c} dr_{v}\\ dr_{a}\\ dr_{av} \end{array}\right]=\left[\begin{array}{c} \frac{1}{1+e^{-sl_{av}\left(th_{av}-dv_{av}\right)}}\times \frac{1}{1+e^{-sl_{u}\left(dv_{v}-dv_{a}\right)}}\\ \frac{1}{1+e^{-sl_{av}\left(th_{av}-dv_{av}\right)}}\times \frac{1}{1+e^{-sl_{u}\left(dv_{a}-dv_{v}\right)}}\\ \frac{1}{1+e^{-sl_{av}\left(dv_{av}-th_{av}\right)}} \end{array}\right] \end{array}$$

*th*_*av*_ is the tunable threshold for the similarity measure above which we perceive the two signals as coming from the same object and below which we can differentiate the cause of the two signals. *sl*_*av*_ and *sl*_*u*_ are function parameters which determine the speed and confidence of the transition between alternative decisions.

The decision result controls the communication of the plan representations from the plan layer, *PL*, to the execution layer, *EX*. Accordingly, the plan representations in *EX* are governed by:

(15) $$\begin{array}{lll} \vec{EX\text{\_}V}\left(t\right) & = & \left[\begin{array}{c} \;ech_{exv}\\ \;ecv_{exv} \end{array}\right]=\;dr_{v}\;\times \;\left[\begin{array}{c} \;ech_{plv}\\ \;ecv_{plv} \end{array}\right] \end{array}$$

(16) $$\begin{array}{lll} \vec{EX\text{\_}A}\left(t\right) & = & \left[\begin{array}{c} \;ech_{exa}\\ \;ecv_{exa} \end{array}\right]=\;dr_{a}\;\times \;\left[\begin{array}{c} \;ech_{pla}\\ \;ecv_{pla} \end{array}\right] \end{array}$$

(17) $$\begin{array}{lll} \vec{EX\text{\_}AV}\left(t\right) & = & \;\left[\begin{array}{c} \;ech_{exav}\\ \;ecv_{exav} \end{array}\right]=\;dr_{av}\;\times \;\left[\begin{array}{c} \;ech_{plav}\\ \;ecv_{plav} \end{array}\right] \end{array}$$

$\vec{DR}$ implements the decision concerning which plan drives the gaze-shift. This is applied by selective inhibition of plan representations in the execution layer (*EX*). *EX* plan representations are selectively inhibited to determine the winning plan. Here, this is shown by the multiplicative effect of the corresponding $\vec{DR}$ component. Such functionality can be neurophysiologically realized by an inhibitory connection from a neural population representing $\vec{DR}$ to the neural populations representing the execution layer (*EX*) plans (Redgrave et al., 1999; Sajad et al., 2015).

## Results

Psychophysicists record the observable behavior of subjects during experiments. However, the neurocognitive internal system underlying the behavior is not accessible to the psychophysicist. For example, for causal inference studies in cross-modal spatial localization, the “report of sameness” is the only measureable behavior, while the whole host of internal mechanisms, e.g., sensory representations, working memory and decision making units, which are responsible for the behavior are not measurable. In this paper we propose a model of the internal cognitive system underlying the implementation of such tasks. In this section: (1) we verify our model against the limited number of psychophysical studies of causal inference during cross-modal spatial localizations which systematically varied both the spatial and temporal features (Slutsky and Recanzone, 2001; Wallace et al., 2004). We do so (in Section Inference of a Unique Cause for Cross-Modal Stimuli) by comparing our model's output with the only measureable behavior “report of sameness” in such experiments. (2) At this stage, we have verified the ability of the model to reproduce the human behavior when the spatial and temporal configurations of the cross-modal stimuli are varied. We then look into the internal system by illustrating the dynamics of the decision variable and decision result when we change the spatial (Section Effect of Spatial Disparity) or temporal (Section Effect of Temporal Disparity) disparities between the stimuli. (3) We then use the model to predictively simulate the human behavior in some novel situations where experimental evidence is not yet available. We first simulate what happens when the reliability of the stimuli vary, when separate sources are perceived (Section Effect of Stimulus Reliability). Then we will illustrate how accumulation of evidence through exposure of the model to temporally extended stimulus presentations may change the decision (Section Effect of Evidence Accumulation).

### Inference of a unique cause for cross-modal stimuli

The percentage of the times that an audio-visual stimulus is judged as arising from a unique cause varies with the spatial and temporal features of the stimuli (Slutsky and Recanzone, 2001; Wallace et al., 2004). Slutsky and Recanzone (2001) kept the position, duration, and onset of the auditory stimulus fixed, and varied the onset and position of the visual stimulus and found how this report of unique cause changes. They found that a unique cause was elicited for small temporal disparities even at large spatial disparities, and also for large temporal disparities for small spatial disparities (Slutsky and Recanzone, 2001).

**Figure 2:**
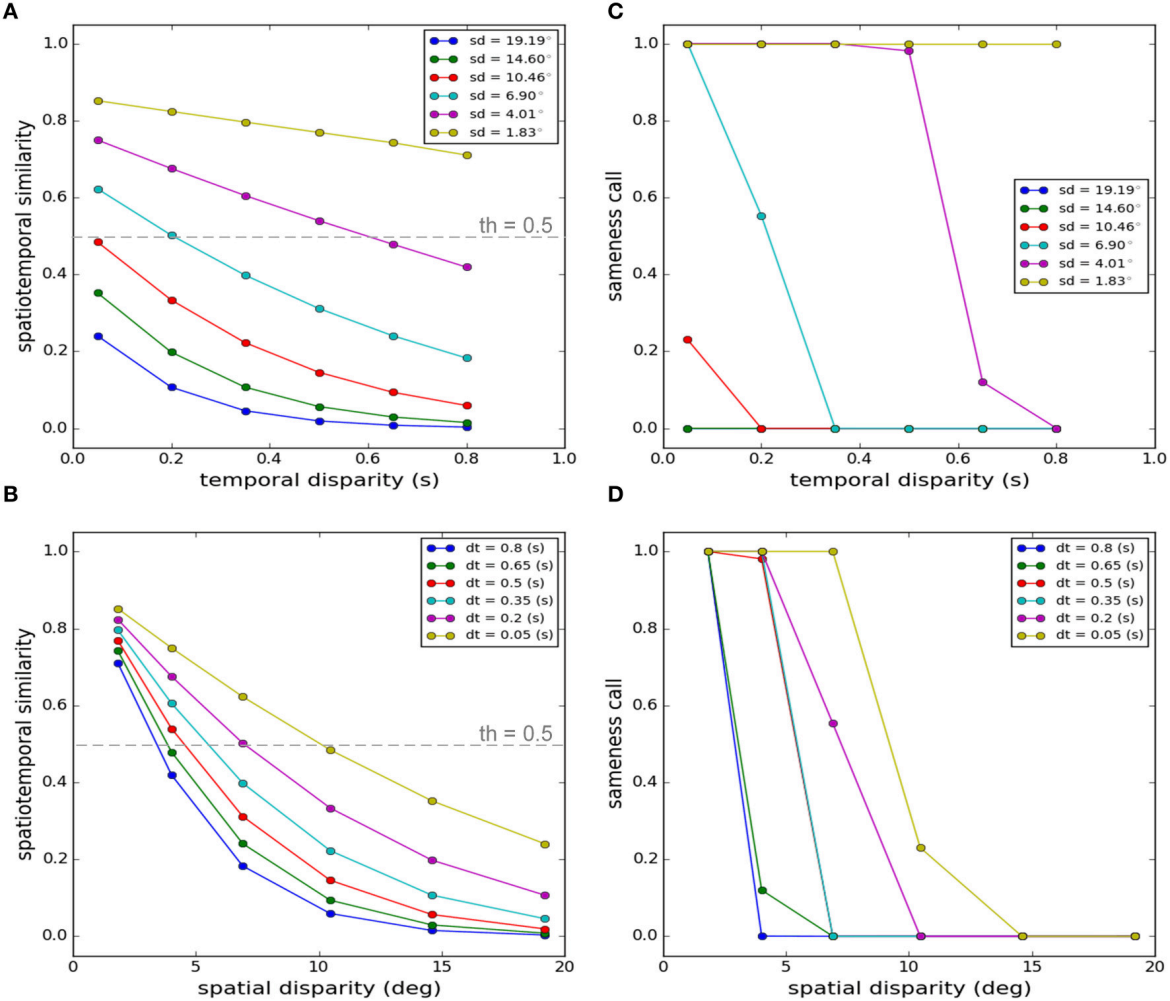
**Spatiotemporal similarity measure as the criterion for the decision on the uniqueness of the cause**. Here we replicate a task where participants were asked to report if two cross-modal stimuli emanated from a unique cause (Slutsky and Recanzone, 2001). While all features of the auditory stimulus were kept fixed, they systematically varied the spatial position and the onset time of the visual stimulus and studied how the sameness report changes. **(A)** Spatiotemporal similarity measure as a function of temporal disparity for different spatial disparities. **(B)** Spatiotemporal similarity measure as a function of spatial disparity for different temporal disparities. **(C)** Sameness call as a function of temporal disparity for different spatial disparities. **(D)** Sameness call as a function of spatial disparity for different temporal disparities. The values “1” and “0” for the sameness call indicate the same source and separate sources respectively. The symbols “sd” and “dt” indicate the spatial (degrees) and temporal disparities (seconds) respectively. The gray dashed lines in **(A,B)** indicate the threshold applied to the similarity measure.

Figure 2 shows the output of our model when stimulus parameters are varied in the same way as Slutsky and Recanzone (2001). Our proposed criterion for this decision is the measure of spatiotemporal similarity. This measure is shown as a function of temporal disparity for different spatial disparities in Figure 2A and as a function of spatial disparities for different temporal disparities in Figure 2B. The decision is made by applying a threshold (set to 0.5 throughout all of our simulations) function to the similarity measure: if it is above threshold, the decision is that there is a unique cause, if it is below threshold the decision is that there are separate causes. The results of this decision are shown in Figure 2C as a function of temporal disparity for different spatial disparities and in Figure 2D as a function of spatial disparities for different temporal disparities.

The average percentage of the reports of a unique cause, among a number of participants and through multiple trials, changing by the spatial and temporal disparities, follow a meaningful pattern, as experimentally observed (Slutsky and Recanzone, 2001). This pattern is closely captured by the trends produced by our model which infers the causal structure based on the spatiotemporal similarity. Unique cause is predicted for a wide range of temporal disparities if the spatial disparity is very small, as shown in Figures 2A,C for a spatial disparity of 1.83° (ventriloquism effect). The “sameness call” changes at some point for most spatial disparities if the temporal disparity becomes greater than threshold. Similarly, the “sameness call” changes for a given temporal disparity if the spatial disparity exceeds some threshold. Thus, although we did not tinker extensively with our model parameters to exactly match the experimental results quantitatively, we conclude that the model replicates the key results and principles of the published experiment.

### Effect of spatial disparity

**Figure 3:**
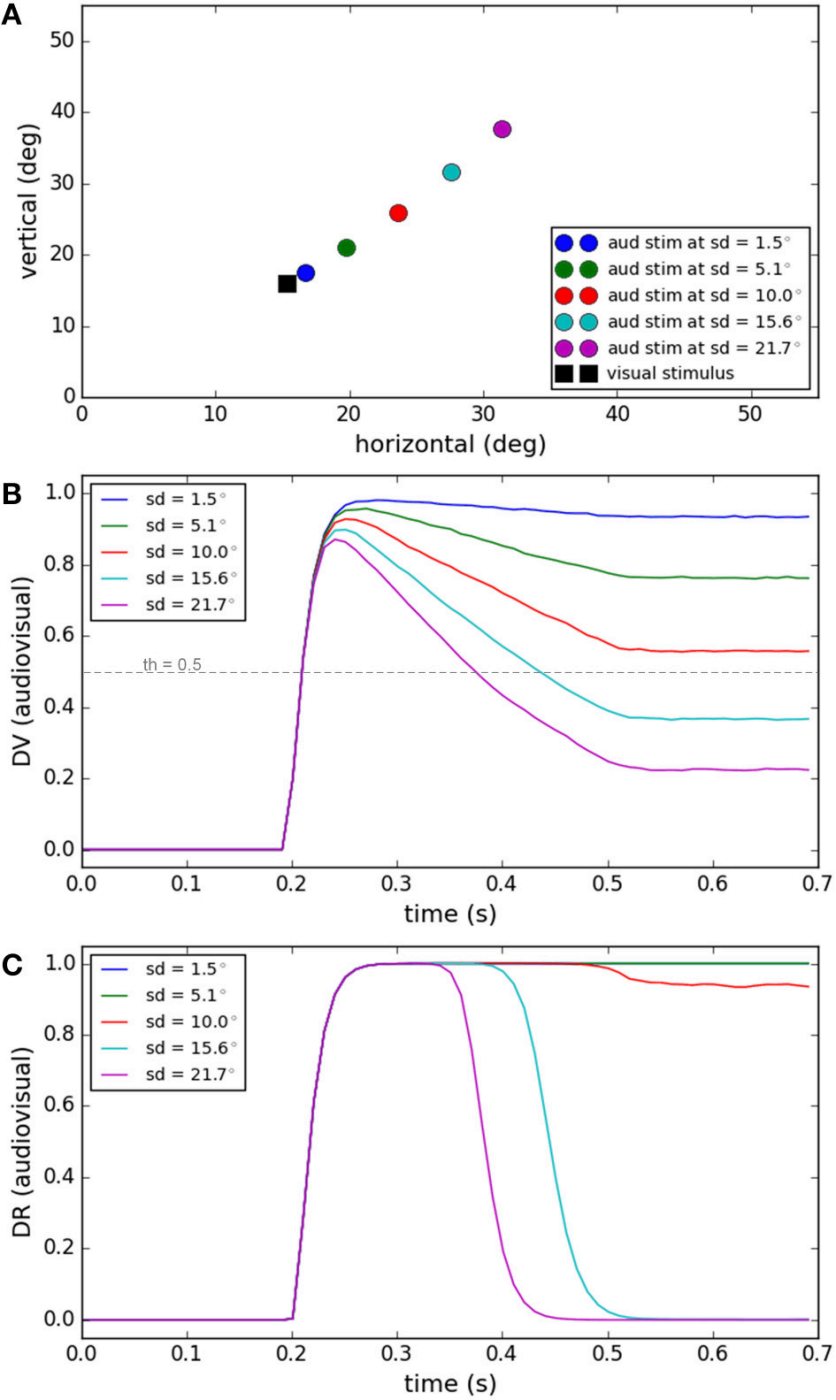
**Effect of spatial disparity of cross-modal stimuli on target selection**. Five different conditions have been considered (illustrated by color coding). The spatial and temporal features of the visual stimulus and the temporal features of the auditory stimulus are fixed for all conditions. The spatial position of the auditory stimulus changes in each condition. **(A)** The spatial position of the visual stimulus and the five different spatial positions of the auditory stimulus, in the five conditions, are shown. **(B)** The multisensory component of the decision variable is shown for different conditions as a function of time. It changes based on the spatial disparity of the stimuli in each condition. The unisensory components do not change. **(C)** The multisensory component of the decision result is shown for the different conditions. It is one for smaller spatial disparities (indicating a common cause) and changes to zero (indicating separate causes) when the spatial disparity exceeds the threshold (shown as a dashed line in **B**).

Spatial proximity is one of the features used to judge whether or not two signals have a common source (Hairston et al., 2003; Wallace et al., 2004). Figure 3 shows the performance of our model for a task in which visual and auditory stimuli have the same onset time (0.2 s) and duration (0.3 s). While the position of the visual stimulus was fixed, the position of the auditory stimuli was varied systematically (spatial disparities from 1.5 to 21.7°, Figure 3A). The end behavior, “sameness call,” of our model for this task has already been validated by experimental results in Section Inference of a Unique Cause for Cross-Modal Stimuli, the yellow lines (very low temporal disparity) in Figures 2B,D, and we want to show the internal dynamics here. Figure 3B shows the similarity measure, represented in the multisensory dimension of the decision variable, for the five spatial disparities. Figure 3C shows the “sameness call,” represented in the multisensory dimension of the decision result, for each spatial disparity. For a fixed temporal structure, the similarity measure decreases when the spatial distance increases. There is a point, around 10° of spatial distance for this case, where the decision about the uniqueness of the cause changes. Our model proposes that the reason is that the similarity measure drops below threshold, and when this happens the unisensory plan with the higher saliency wins and is executed (not shown here). These simulations show how the temporal evolution of the internal system is influenced when the spatial disparity between cross-modal stimuli varies, sometimes leading to a change in decision through time (sd = 15.6 or 21.7° here).

### Effect of temporal disparity

**Figure 4:**
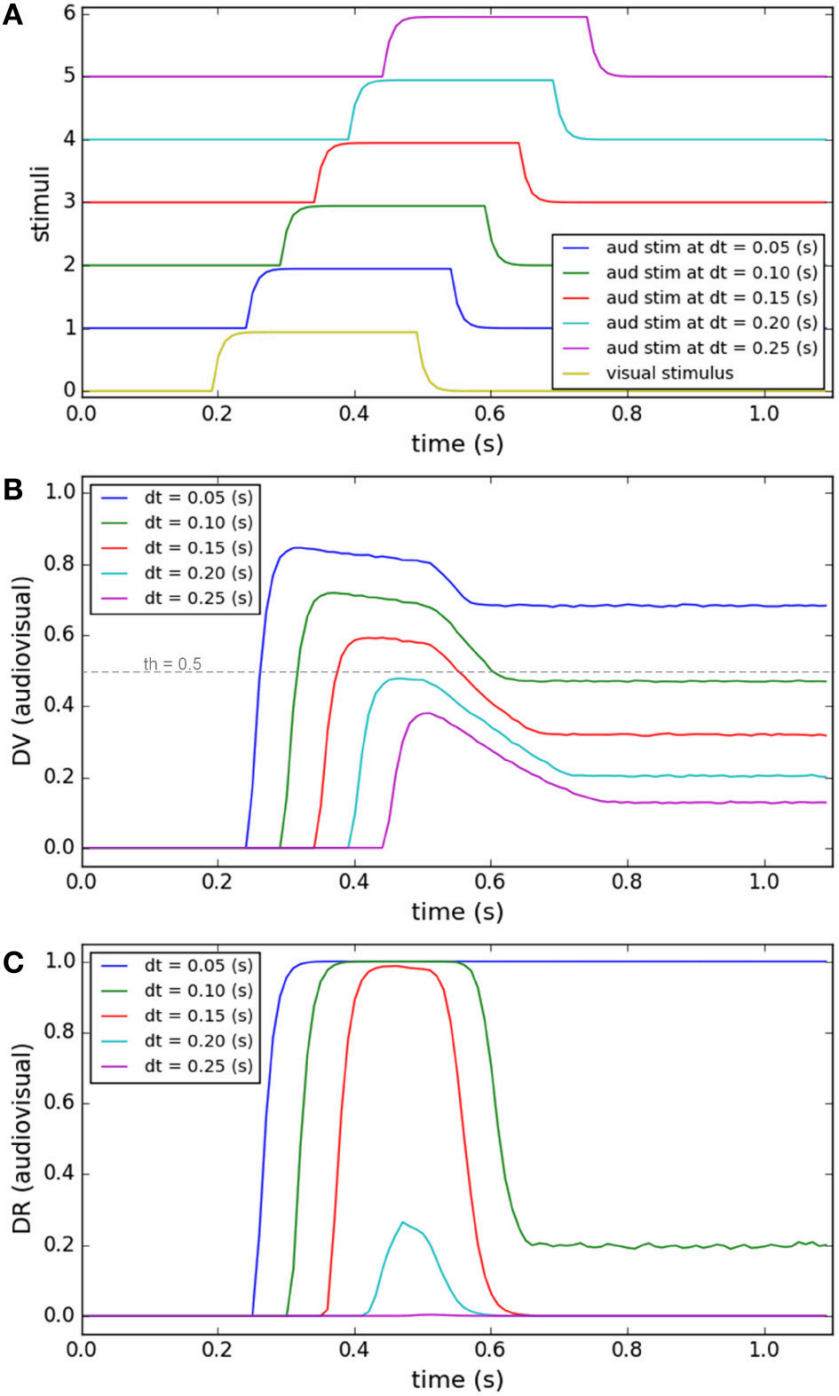
**Effect of temporal disparity of cross-modal stimuli on target selection**. Five different conditions are considered (illustrated by color coding). The spatial and temporal features of the visual stimulus and the spatial features of the auditory stimulus are fixed for all conditions. The onset time of the auditory stimulus varies from 0.25 to 0.45 s. **(A)** The temporal profile of the visual stimulus (lower curve, fixed) and the auditory stimulus (5 upper curves, changing). **(B)** The multisensory component of the decision variable is shown for different conditions as a function of time. It changes for different conditions based on the temporal disparity of the stimuli in each condition. The unisensory components (not shown) don't change for different conditions. **(C)** The multisensory component of the decision result is shown for different conditions. It is “1” (single source) for smaller temporal disparities and changes to “0” (multiple sources) when the temporal disparity exceeds the threshold (shown as a dashed line in **B**).

Temporal disparity is another feature that contributes to the decision about the sameness of the cause of the signals (Wallace et al., 2004; Chen and Vroomen, 2013). In Figure 4 we show the simulations of our model under a task in which the visual and auditory stimuli have fixed positions close to each other. The duration of the auditory stimulus and visual stimulus are fixed (0.3 s). As shown in Figure 4A, while the onset time of the visual stimulus is fixed (0.2 s), the onset time of the auditory stimulus varies systematically (from 0.25 to 0.45 s). The end behavior, “sameness call,” of our model for this task has already been validated by experimental results in Section Inference of a Unique Cause for Cross-Modal Stimuli, the blue lines (spatial disparity around 7°) in Figures 2A,C, and we want to show the internal dynamics here. Figure 4B shows the similarity measure, represented in the multisensory dimension of the decision variable, for five temporal disparities. Figure 4C shows the sameness calls, represented in the multisensory dimension of the decision result. For a fixed spatial structure, the similarity measure decreases when the temporal disparity increases. There is a point, around 0.1 s of temporal disparity for this case, that the decision about the uniqueness of the cause changes. Based on the mechanism proposed in our model, the change in the sameness call occurs when the spatiotemporal similarity between the stimuli falls below threshold which leads to the more reliable of the unisensory plans to win (not shown here). These simulations show how the temporal evolution of the internal system is influenced when the temporal disparity between cross-modal stimuli varies, sometimes leading to a change in decision through time [dt = 0.1(s) or 0.15(s) here].

### Effect of stimulus reliability

**Figure 5:**
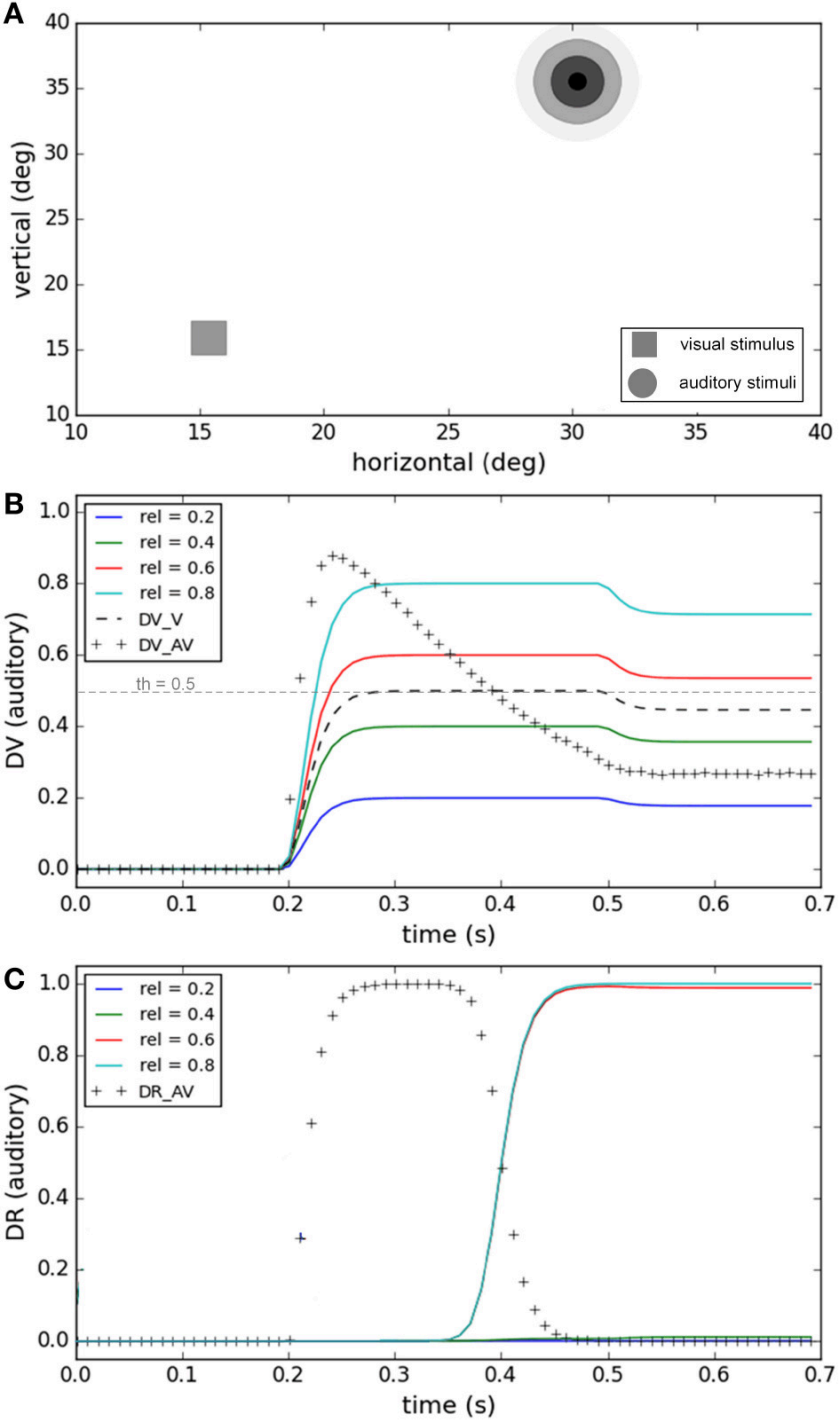
**Effect of the reliability of the unimodal stimuli on target selection**. Four different conditions are considered (illustrated by color coding) The spatiotemporal features of both stimuli are fixed and are chosen such that the similarity measure is always small enough that separate causes are distinguished in all conditions. **(A)** The visual stimulus with fixed reliability is shown by a square. The auditory stimulus with varying reliability is illustrated by concentric circles of different levels of blur. **(B)** The decision variable is shown for different conditions as a function of time. The visual (thick dashed line) and multisensory components (line of crosses) are the same for all conditions. The auditory component (solid colored lines) varies between different conditions based on the reliability of the auditory stimuli, as shown in **(A)**. **(C)** The decision result for the auditory component is shown for different conditions as a function of time. The multisensory component (line of crosses) is the same for all conditions. The auditory component is unity when the reliability of the auditory stimulus is higher than the visual stimulus and changes to zero when the auditory stimulus is more reliable than the visual stimulus. The visual component of decision changes in the opposite way.

For the cases in which there is a large spatiotemporal misalignment between the two stimuli, human subjects often infer that two separate sources exist (Chen and Vroomen, 2013; Ursino et al., 2014) and plan a gaze-shift toward the more salient of the two separate signals. In Figure 5 we show the performance of our model under a task in which the visual and auditory stimuli are far from each other in space. The spatiotemporal structure is fixed, and the reliability of the visual stimulus (0.5) is also not changing. The variable factor is the reliability of the auditory stimulus which is changing from unreliable (0.2) to highly reliable (0.8) in four conditions (Figure 5A). Figure 5B shows how the decision variable changes through time for the four conditions. The multisensory (crosses) and visual dimensions (dashed lines) of the decision variable are the same for all conditions, but the auditory dimension is different under each condition because the reliability of auditory stimulus changes. Figure 5C shows result of the auditory plan winning, represented in the auditory dimension of the decision result, for each condition. At the time 0.4 (s) the multisensory component of the decision variable drops below the threshold (Figure 5B), the multisensory component of the decision result changes from zero to one, the unisensory component of the decision result (corresponding to the more reliable stimulus) changes from one to zero, and two separate sources are recognized. When the reliability of visual stimulus is higher than the auditory stimulus the visual plan wins, and if it is lower the auditory plan wins. These simulations show how the temporal evolution of the internal system is influenced when the reliabilities of stimuli vary, leading to selection of the more reliable stimulus as the goal.

### Effect of evidence accumulation

**Figure 6:**
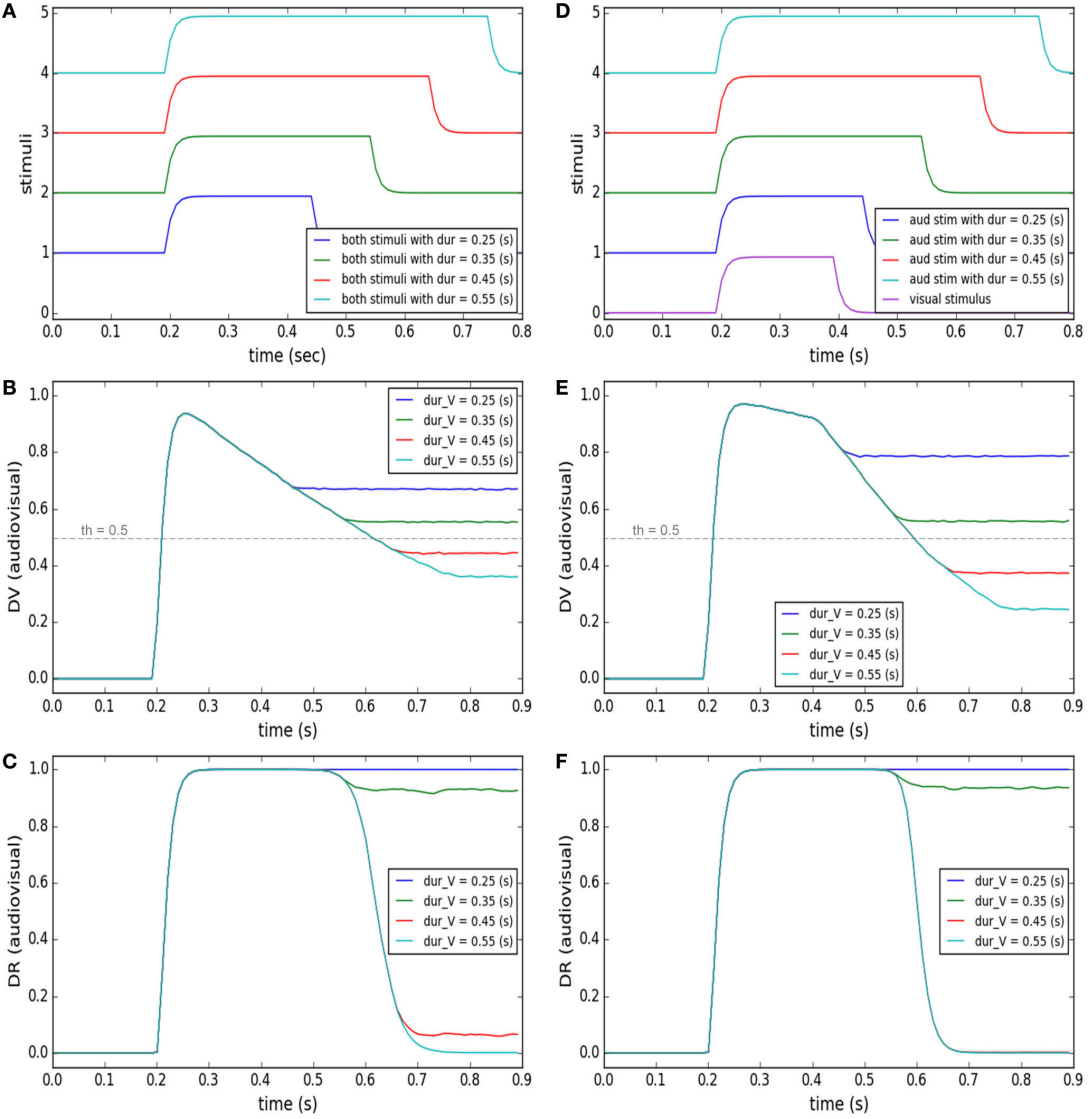
**Effect of accumulation of evidence about cross-modal stimuli on changing target selection decision**. In each of the columns **(A–F)** four different conditions are considered (illustrated by color coding). In the left column, the temporal features of the two stimuli are exactly the same. The stimuli are presented at a fixed, small spatial distance from each other in all conditions. Only the duration of presentation of the stimuli varies for the different conditions (from 0.25 to 0.55 s). In the right column, the spatial and temporal features of the visual stimulus are fixed (purple curve in **D**). The two stimuli have a same onset time (0.2 s) and are presented at a fixed distance from each other, in all conditions. However, the duration of presentation of the auditory stimulus changes from 0.25 to 0.55 (s) (curves 1–4 in **D**). **(A,D)** temporal profiles of the stimuli. **(B,E)** The multisensory component of the decision variable is shown for different conditions as a function of time. It changes for different conditions. The unisensory components do not change for different conditions (not shown). The threshold value is shown as a horizontal dashed line. **(C,F)** The multisensory component of the decision result is shown for the different conditions. It is initially unity (common cause) first when the stimuli appear. However, it may change to zero (separate causes) if and when enough evidence has accumulated to support the existence of two separate causes.

Accumulation of evidence may lead the decision to lean toward an alternative category other than the currently preferred category (Gold and Shadlen, 2007). This has been observed in many oculomotor tasks, for instance, in “anti-saccade” task where the subjects, by default, would plan a saccade toward the presented target, unless some instructive cue commands them to plan a saccade in the mirror opposite direction to the target, in contrast to the default (Everling and Fischer, 1998; Munoz and Everling, 2004). Another example is the “saccade countermanding” task where the subject, by default, has to make a saccade toward the visual target, unless some cue instructs it to stop the motor plan and keep fixating (Hanes and Schall, 1995; Schall et al., 2000). In our case, when stimuli from multiple modalities are presented, we postulate that the default is to assume a common cause for them. This default can be changed to another decision, i.e., separate causes, by accumulation of evidence over time. This concept has been materialized in our model by the development of the similarity measure and its effect on the decision result. We illustrate this concept in two tasks shown in the left and right columns of Figure 6.

The left column shows the model's predictions for a case where two stimuli are presented at fixed positions close to each other. As illustrated in Figure 6A, the duration of time that the stimuli are present is varied (from 0.25 to 0.55 s). Figure 6B shows the similarity measure, represented in the multisensory dimension of the decision variable (*dv*_*av*_), and Figure 6C shows the sameness call, represented in the multisensory dimension of the decision result (*dr*_*av*_), developing across time. When the two stimuli are presented briefly and at the same time, they are perceived as belonging to a common source even if they are not presented at exactly the same position in space. But for the same spatial configuration, if the duration of stimulus presentation increases, the similarity measure decreases. There is a point, around 0.4 s of presentation duration for this case, that the decision about the uniqueness of the cause changes.

The right column shows the model's prediction for a case where one stimulus appears briefly but the other stimulus might stay on for a longer time. The auditory and visual stimuli, presented at fixed positions very close to each other, have the same onset time (0.2 s) but the auditory stimulus is on from 0.05 to 0.35 s longer than the visual stimulus (which has a duration of 0.2 s; Figure 6D). Figure 6E shows the similarity measure, represented in the multisensory dimension of the decision variable, and Figure 6F shows the sameness call, represented in the multisensory dimension of the decision result, developing over time. By extending the presentation duration of one stimulus, while the other is presented only briefly, the similarity measure decreases. Therefore, the sameness decision which was for a common source for shorter durations changes to being for separate sources for longer durations. These examples show that the default decision (that stimuli arise from a common cause) can be altered over a period of time during which evidence accumulates indicating (perhaps) that they are in fact separate. The duration over which evidence needs to accumulate may correspond to the temporal binding window.

## Discussion

In summary, we have proposed a computational model of the cognitive internal system underlying causal inference in spatial localization of cross-modal stimuli. The emerging output of this internal system (report of sameness), not itself, is measurable by psychophysicists. We first showed that our model can replicate the behavioral reports of the perception of a common cause measurable in psychophysical experiments. Having verified the model, we then moved on to illustrate the dynamics of the decision variable and decision result when spatial and temporal features of the stimuli were changing, like the existing tasks. We then showed the system dynamics for novel situations were separate causes would be inferred or when the decision would change from common to separate sources through evidence accumulation. These dynamic simulations may be tested by new experiments that force the subject's report at specific times and see if the decision changes based on the timing of this forced decision.

Importantly, this new model incorporates several novel features that we expect to be valuable for understanding multisensory integration in the real brain. Based on the ability of our model to replicate known behavioral results (References), and contingent on the further verification of our model's new predictions, we propose that (1) the brain's distributed working memory is multisensory and should retain and process the sensory information to perform this task. (2) Separate computational units are required for representing alternative plans (probably in the cortex) whose selective inhibition (perhaps through basal ganglia connections to cortex) implements the result of the decision. (3) A central decision-making unit should exist capable of applying decision rules, and choosing between multiple causal scenarios based on sensory evidence. (4) Our spatiotemporal similarity measure, capturing how similar the spatial and temporal features of the stimuli are, is the criterion for inferring a common cause. In short, we suggest that the real brain incorporates similar features as our model at the computational level. Further, the current computational-level model is constructed in such a way as to provide a potential formal framework for models that generate physiological predictions at the level of single units and networks.

Finally, the model framework that we have proposed here (simulating causal inference from one visual and one auditory stimulus) has the potential to generalize to a number of other, more complex situations where working memory is a limiting factor. For example: (1) one can tackle target selection between more than two stimuli (Schall and Hanes, 1993; Hill and Miller, 2010) by enhancing the capacity of our short-term memory, increasing the number of possible plan representations and the dimensions of the decision variable, and defining a multi-dimensional distance variable. (2) One can address causal inference and integration for other modality combinations like visual/tactile and auditory/tactile (Menning et al., 2005; Katus et al., 2015). (3) One can address a situation where a subject has a prior expectation of where the target would appear (Van Wanrooij et al., 2010). When the target is presented one has a causal inference problem to solve, which is whether or not the presented and expected signals are the same, and whether or not to integrate the internal and sensory representations. (4) One can extend the features of the stimuli to include semantic or emotional values (Robertson, 2003). This requires expansion of our concept of similarity to include the more cognitive and linguistic aspects assigned to the stimuli.

## Author contributions

MD: He was the main contributor to the conception and design of the work and writing it in the form this manuscript; LH: He critically revised the manuscript and added immense intellectual content; JC: He provided constant guidance and discussion all through the conception of the work. He also provided the financial support for this work.

## Funding

This work was supported by the NSERC CREATE CAN-ACT Program, the CIHR Operating Program, and the JC's Canada Research Chair.

### Conflict of interest statement

The authors declare that the research was conducted in the absence of any commercial or financial relationships that could be construed as a potential conflict of interest.

## Nomenclature

$$\begin{array}{ll} \vec{V}\left(t\right)=\;\left[\begin{array}{c} ext_{v}\\ \;rel_{v}\\ \;ech_{v}\\ \;ecv_{v} \end{array}\right]:visual\;signal\;\\ = & \left[\begin{array}{c} existence\\ reliability\\ horizontal\;position\\ vertical\;position \end{array}\right] \end{array}$$

$$\begin{array}{lll} \vec{A}\left(t\right) & = & \;\left[\begin{array}{c} ext_{a}\\ \;rel_{a}\\ \;ech_{a}\\ \;ecv_{a} \end{array}\right]:auditory\;signal\;\\ = & \left[\begin{array}{c} existence\\ reliability\\ horizontal\;position\\ vertical\;position \end{array}\right] \end{array}$$

$$\begin{array}{ll} \vec{M}\left(t\right)=\;\left[\begin{array}{c} lk_{mv}\\ \;rel_{mv}\\ \;ech_{mv}\\ \;ecv_{mv}\\ \begin{array}{c} \;lk_{ma}\\ \;rel_{ma}\\ \;ech_{ma}\\ \;ecv_{ma} \end{array} \end{array}\right]:multisensory\;memory\;\\ = & \left[\begin{array}{c} leak\\ reliability\\ horizontal\;position\\ vertical\;position\\ \begin{array}{c} leak\\ reliability\\ horizontal\;position\\ vertical\;position \end{array} \end{array}\right] \end{array}$$

$$\begin{array}{ll} \vec{PL\text{\_}V}\left(t\right)=\;\left[\begin{array}{c} sal_{plv}\\ \;ech_{plv}\\ \;ecv_{plv} \end{array}\right]:visual\;plan\;in\;plan\;layer\;\\ = & \left[\begin{array}{c} saliency\\ horizontal\;position\\ vertical\;position \end{array}\right] \end{array}$$

$$\begin{array}{ll} \vec{PL\text{\_}A}\left(t\right)=\;\left[\begin{array}{c} sal_{pla}\\ \;ech_{pla}\\ \;ecv_{pla} \end{array}\right]:auditory\;plan\;in\;plan\;layer\;\\ = & \left[\begin{array}{c} saliency\\ horizontal\;position\\ vertical\;position \end{array}\right] \end{array}$$

$$\begin{array}{ll} \vec{PL\text{\_}AV}\left(t\right)=\;\left[\begin{array}{c} sal_{plav}\\ \;ech_{plav}\\ \;ecv_{plav} \end{array}\right]:multisensory\;plan\;in\;plan\;layer\;\\ = & \left[\begin{array}{c} saliency\\ horizontal\;position\\ vertical\;position \end{array}\right] \end{array}$$

$$\begin{array}{ll} \vec{EX\text{\_}V}\left(t\right)=\;\left[\begin{array}{c} ech_{exv}\\ \;ecv_{exv} \end{array}\right]:visual\;plan\;in\;execution\;layer\;\\ = & \left[\begin{array}{c} horizontal\;position\\ vertical\;position \end{array}\right] \end{array}$$

$$\begin{array}{ll} \vec{EX\text{\_}A}\left(t\right)=\;\left[\begin{array}{c} ech_{exa}\\ \;ecv_{exa} \end{array}\right]:auditory\;plan\;in\;execution\;layer\;\\ = & \left[\begin{array}{c} horizontal\;position\\ vertical\;position \end{array}\right] \end{array}$$

$$\begin{array}{ll} \vec{EX\text{\_}AV}\left(t\right)=\;\left[\begin{array}{c} ech_{exav}\\ \;ecv_{exav} \end{array}\right]:multisensory\;plan\;in\;execution\;layer\;\\ = & \left[\begin{array}{c} horizontal\;position\\ vertical\;position \end{array}\right] \end{array}$$

$$\begin{array}{l} DIST\left(t\right)=\;\left[dist\right]:spatial\;distance \end{array}$$

$$\begin{array}{ll} \vec{DISP}\left(t\right)=\;\left[\begin{array}{c} lk_{disp}\\ disp \end{array}\right]:spatiotemporal\;disparity\;\\ = & \left[\begin{array}{c} leak\\ spatiotemporal\;disparity \end{array}\right] \end{array}$$

$$\begin{array}{l} SIM\left(t\right)=\left[sim\right]:spatiotemporal\;similarity \end{array}$$

$$\begin{array}{ll} \vec{DV}\left(t\right)=\;\left[\begin{array}{c} dv_{v}\\ dv_{a}\\ dv_{av} \end{array}\right]:decision\;variable\;\\ = & \left[\begin{array}{c} bid\;of\;visual\;plan\\ bid\;of\;auditory\;plan\\ bid\;of\;audiovisual\;plan \end{array}\right] \end{array}$$

$$\begin{array}{ll} \vec{DR}\left(t\right)=\;\left[\begin{array}{c} dr_{v}\\ dr_{a}\\ dr_{av} \end{array}\right]:decision\;result\\ = & \left[\begin{array}{c} result\;of\;visual\;plan\\ result\;of\;auditory\;plan\\ result\;of\;audiovisual\;plan \end{array}\right] \end{array}$$
